# ABBaH: Activity Breaks for Brain Health. A Protocol for a Randomized Crossover Trial

**DOI:** 10.3389/fnhum.2020.00273

**Published:** 2020-07-14

**Authors:** Emerald G. Heiland, Örjan Ekblom, Olga Tarassova, Maria Fernström, Coralie English, Maria M. Ekblom

**Affiliations:** ^1^The Swedish School of Sport and Health Sciences (GIH), Stockholm, Sweden; ^2^School of Health Sciences and Priority Research Centre for Stroke and Brain Injury, University of Newcastle, Newcastle, NSW, Australia

**Keywords:** physical activity breaks, sedentary, cerebral blood flow, cognitive function, fNIRS

## Abstract

**Introduction**: Extended periods of sitting may have detrimental effects on brain health. However, the effects of breaking up prolonged sedentary periods with frequent, short physical activity bouts on mechanisms to improve brain health remain unclear. Therefore, this study aims to investigate the immediate effects of uninterrupted sitting and frequent, short bouts of physical activity on cerebral blood flow and cognitive function in the prefrontal cortex in middle-aged adults.

**Methods**: This is a protocol article to describe a randomized crossover study. We will collect data from 13 healthy adults, aged between 40 and 60 years old, with a body mass index <35 kg/m^2^. Participants will be required to come into the laboratory on three occasions, sit for 3 h, and perform a different type of break for 3 min every 30 min at each visit in a random order, being either: (1) a social break; (2) brisk walk on a treadmill; or (3) simple resistance activities. Before and after each experimental condition, cerebral blood flow (primary outcome) will be measured using functional near-infrared spectroscopy (fNIRS), with short-separation channels, and working memory (1-, 2-, and 3-back on the computer) will be assessed. The following additional secondary outcomes will be collected: psychological factors (questionnaires); arterial stiffness; salivary cortisol levels; and blood glucose levels.

**Conclusion**: The results from this randomized crossover study will determine the effects of uninterrupted sitting and frequent, short bouts of physical activity on cerebral blood flow and cognitive performance. Publication of this study protocol emphasizes the importance of registration and publication of protocols in the field of sedentary behavior research.

## Introduction

Sedentary behavior is gaining more attention owing to its association with increased risk of cardiovascular diseases and early mortality (Biswas et al., 2015; Patterson et al., 2018; Ekelund et al., 2019), and potential adverse effects on cognitive and mental health (Voss et al., 2014; Wheeler et al., 2017). Understanding the neurophysiological consequences of sedentary behavior is important considering that adults spend about two-thirds of their waking time and the majority of working hours in long periods of uninterrupted sitting (Parry and Straker, 2013).

Physical activity is known to promote healthy brain functions (i.e., cognitive and mental health; Angevaren et al., 2008; Hillman et al., 2008; Gomez-Pinilla and Hillman, 2013); and a candidate mechanism by which this can occur is through increased cerebral blood flow (Ogoh and Ainslie, 2009), particularly in the prefrontal cortex (Herold et al., 2018).

In light of the negative effects of sedentary behavior on vascular outcomes, it is possible that prolonged sitting may have adverse effects on brain health by reducing cerebral blood flow. However, sedentary behavior’s effects on cerebral blood flow, the mitigating effects of physical activity breaks, and what types of physical activity breaks remain unknown. A few studies, in younger- or older-aged populations, have demonstrated immediate improvements in cerebral blood flow from a single exercise session (Chang et al., 2012; Herold et al., 2018; Ji et al., 2019), whereas the influence of breaking up a sitting day with frequent (e.g., 30 min), short (e.g., 3 min) bouts of physical activity on increasing cerebral blood flow is less clear. Using a surrogate measure of cerebral blood flow (middle cerebral artery blood flow velocity), one study of healthy desk workers found immediate increases in blood flow velocity after short, frequent bouts of walking compared with uninterrupted sitting for 4 h (Carter et al., 2018). Also, in a study of older adults, middle cerebral artery blood flow velocity increased over a day of interrupted sitting with light-intensity walking breaks for 3 min every 30 min, after performing a 30-min moderate-intensity walk in the morning before the prolonged sitting time (Wheeler et al., 2019). However, another study of young adults found a reduction in cerebral blood flow and cognition after short, frequent bouts of calf raises over 3 h, compared to uninterrupted sitting for 3 h (Stoner et al., 2019). Finally, a study of 19 overweight and obese adults demonstrated improved fatigue after short, frequent bouts of light-intensity walking, which may be explained by improved cerebrovascular regulation, although this was not directly measured (Wennberg et al., 2016). Thus, results from previous studies are inconsistent and emphasize the need for further studies. Middle-aged adults may be at particular risk as low cerebral blood flow may contribute to future cognitive decline (Bertsch et al., 2009).

Moderate-to-vigorous intensity physical activity levels are often not attainable by most people and knowing the negative health effects of sedentary behavior, strategies to increase physical activity are needed that are manageable for long term (Dunstan et al., 2018). Earlier studies have demonstrated positive effects of frequent (e.g., 30 min), short (e.g., 3 min) breaks of physical activity on glycemic control (Dunstan et al., 2012; Dempsey et al., 2017), arterial health (Carter et al., 2019), middle cerebral artery velocity (Wheeler et al., 2019), fatigue (Wennberg et al., 2016), and cognitive performance (Wheeler et al., 2020). However, these feasible breaks have yet to be studied concerning simultaneously measured cerebral blood flow and cognitive performance.

Functional-near infrared spectroscopy (fNIRS) is an emerging neuroimaging technique that allows for accurate measurement of cerebral blood flow, such as in the prefrontal cortex (Herold et al., 2018). Other neuroimaging methods to measure cerebral blood flow, such as positron emission tomography and functional magnetic resonance imaging (fMRI) are cumbersome, due to the size and expense of the equipment, the need for participants to be supine, and high sensitivity to movement artifacts. By contrast, fNIRS is a more ecologically valid method to study cerebral blood flow, as measurement can be performed in various postures and settings. It is portable, non-invasive, cost-effective, and accurate when compared with fMRI measures (Huppert et al., 2006). One concern with fNIRS is that changes in superficial (i.e., extra-cerebral) blood flow may confound measures. Including short-separation channels in fNIRS systems allows the direct measurement of extra-cerebral blood flow thereby eliminating this as a confounder (Yücel et al., 2015), yet has rarely been used to date, but will be used in this study. In middle- and older-aged adults, vascular health and in particular arterial stiffness may moderate the effect of physical activity on cerebral blood flow (Elias et al., 2009; Tarumi et al., 2013) and is therefore also important to investigate.

The overall aim of this study is to explore the effect of uninterrupted sitting and frequent, short bouts of physical activity during a prolonged period of sitting on cerebral blood flow and cognitive function.

Our *primary* research question is:

1 In middle-aged adults, what are the acute (immediate) effects of uninterrupted sitting and frequent, short activity breaks during 3 h of sitting on cerebral blood flow in the prefrontal cortex?
  Our *secondary* research questions are:
2 In middle-aged adults, what are the acute (immediate) effects of uninterrupted sitting and frequent short activity breaks during 3 h of sitting on:
  (a) Cognitive performance?
  (b) Psychological factors (stress, mood, alertness, and sleepiness)?
  (c) Post-prandial blood glucose?
3 Does vascular health (arterial stiffness) attenuate the effects of activity breaks on cerebral blood flow?
  Based on previous research, we hypothesize that:
  (a) Changes in neural activity-related prefrontal cortex cerebral blood flow (hereafter termed cerebral blood flow) from pre-test to post-test will be greater following the conditions with frequent, short activity breaks compared with the uninterrupted sitting condition;
  (b) Cerebral blood flow will be greater post-intervention compared to pre-intervention for the frequent, short activity breaks conditions, but not for the uninterrupted sitting condition;
  (c) Improvements in cognitive performance will be larger in magnitude in the frequent, short activity breaks conditions compared with the uninterrupted sitting condition;
  (d) The frequent, short activity break conditions will lead to improvements in psychological factors, compared with the uninterrupted sitting condition;
  (e) Glucose levels will not differ between the conditions;
  (f) Arterial stiffness will temporarily decrease immediately after the frequent, short activity break conditions (compared to pre-test levels), but will not change after the uninterrupted sitting condition; and
  (g) Arterial stiffness will moderate the effect of frequent, short activity breaks on cerebral blood flow, such that participants with lower arterial stiffness at baseline will have greater cerebral blood flow after the frequent, short activity break conditions compared with participants with higher arterial stiffness.

## Materials and Methods

### Design

A crossover randomized 3-condition experimental trial will be carried out with a target sample size of 13 participants. All study procedures will occur at the Swedish School of Sport and Health Sciences (GIH) in Stockholm, Sweden. Participants will be recruited from the general Swedish population using social media, GIH’s homepage, and newspaper advertisements. As well, through the Swedish Armed Forces’ personnel magazine and word of mouth. Participants will initially attend a familiarization session for the collection of demographic data, fitness testing, and acquaintance with experimental procedures. Each participant will undergo 3-h experimental conditions in random order, with a minimum washout period of 4 days between each of the three visits (Bell et al., 2018). Physical activity, sleep, and glucose levels will be measured for the 24 h before each condition. A computer-generated randomization schedule will be created before study commencement, and participants will be randomized to a condition order at the end of the familiarization session. This trial obtained ethical approval by the Swedish Ethical Review Authority, Stockholm, Sweden (Dnr 2019-00998). Trial registration for experimental studies of this nature is not required but is increasingly being recognized as good practice (English et al., 2020). Therefore, we registered this trial at www.clinicaltrials.gov (NCT04137211) on October 23, 2019, after recruitment began on May 13, 2019.

### Participants

We will include adults 40–60 years old, with a body mass index <35 kg/m^2^. People who have been diagnosed with diabetes, epilepsy, heart failure, stroke, or myocardial infarction, or other circulatory conditions will be excluded. Also, persons receiving current treatment for high blood pressure, sleep disorders, depression, or psychosis.

### Familiarization

Before randomization, participants will come to the laboratory at GIH for a familiarization visit where they will be introduced to all procedures of the study, including a practice of the cognitive tests. Demographic and other data to describe the sample will be collected including age, sex, height, weight, head circumference (to determine the optimal fNIRS cap-size), and a health screening questionnaire. Participants will also undergo an incremental treadmill test to establish their maximal oxygen uptake (VO_2max_) using a standard protocol and the OxyCon Pro metabolic system (Erich Jaeger GmbH, Hochenberg, Germany). The results of the fitness test will be used to determine the intensity of the walking break condition.

### Pre-experimental Day Monitoring

To standardize the 24 h before each test day, participants will be advised to abstain from any physical activity and alcohol intake and will be asked to record details about their physical activity and sleep in a standardized diary. Participants will also wear activity monitors during this period to collect objective data about physical activity (hip-worn actigraph GT3X+ and activPAL micro) and sleep (wrist-worn actigraph GT3X+). Physical activity variables from the actigraph will include total physical activity and percentages of wear time spent in sedentary, light, and moderate-to-vigorous physical activity, according to standardized cut-points (Sasaki et al., 2011; Aguilar-Farias et al., 2014). Physical activity variables from the activPAL micro monitor will include time spent sitting including in prolonged bouts (>20 min), number of breaks from sitting each hour and time spent standing, stepping, and intense stepping. During night time, participants will wear the actigraph GT3X+ on the non-dominant wrist (Cellini et al., 2013). Using the existing Cole-Kripke algorithm (Cole et al., 1992), the following key aspects of sleep will be calculated: time to sleep onset, sleep efficiency, times waking up after sleep onset, and total sleep.

To standardize dietary intake and blood glucose levels, participants will be asked to record their dietary intake for the 24 h before each condition in a standardized food diary and closely match their diet for each pre-condition day. They will also be asked to fast and abstain from caffeine after dinner the night before. Additionally, participants will wear a continuous blood glucose monitor (Abbott FreeStyle Libre sensor) during the 24 h before, and during each experimental condition.

### Experimental Conditions

To minimize physical activity the morning of the visit participants will be transported to the laboratory *via* taxi. On the morning of testing, participants will be asked to provide four saliva samples (for cortisol testing); immediately after waking and at 30, 45, and 60 min thereafter. Once they arrive at the laboratory, pre-condition outcome measures will be collected, followed by an individually standardized breakfast (consisting of maximum two slices of bread with butter and cheese, a bowl of yogurt, a banana, and a glass of water), with no specific time restriction for eating. Participants at their initial visit will be advised to eat their preferred amount, which will be subsequently weighed and recorded. The portion sizes will be then replicated at each visit. The activPAL activity monitor and glucose monitor will be worn during the experimental conditions to monitor sitting time during the test day.

**Figure 1:**
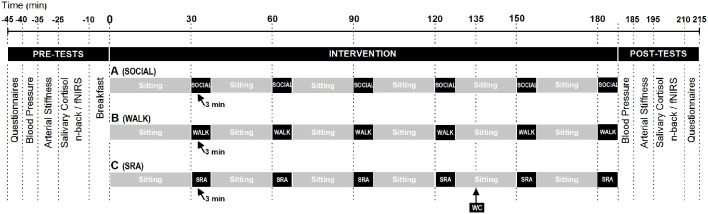
Experimental day procedures of the three conditions after a standardized breakfast. Condition A: 3 h of sitting with 3-min social breaks every 30 min (SOCIAL); Condition B: sitting 3 h with a 3-min bout of brisk walking on a treadmill every 30 min (WALK); Condition C: sitting for 3 h with a 3-min bout of simple resistance activities every 30 min (SRA). WC: toilet break; fNIRS, functional near-infrared spectroscopy.

The three experimental conditions (summarized in Figure 1) are: (A) uninterrupted sitting for 3 h with a 3-min social break every 30 min (SOCIAL); (B) sitting for 3 h with a 3-min bout of moderate-intensity walking on a treadmill every 30 min (WALK), or (C) sitting for 3 h with a 3-min bout of simple resistance activities every 30 min (SRA). During the sitting, participants will be permitted to read a book and drink water, but not use any technological devices or doze off. The SOCIAL break (condition A) will consist of a 3-min chat between the research staff and the participant. The WALK break (condition B) will be performed at moderate intensity (75–80% of maximal heart rate), normalized to individual participants’ VO_2max_ (see Supplementary Material). For the SRA break (condition C), participants will be asked to complete 20-s bodyweight half-squats, 20-s calf raises, and 20-s gluteal contractions and knee raises repeated three times in sequential order by following standardized video instructions (as per Dempsey et al., 2017). At 135 min after the experimental condition commences, participants will be offered a toilet break.

### Outcome Measures

Outcome measures will be collected immediately before and immediately after each experimental condition.

#### Cerebral Blood Flow

The primary outcome measure will be the changes in oxygenated hemoglobin (oxy-Hb) using a non-invasive multi-channel continuous-wave fNIRS instrument (portable NIRSport, 8–8 system, with short-separation channels, NIRx Medizintechnik GmbH, Berlin, Germany). The optodes will be positioned over the prefrontal cortex, with data sampled at 7.81 Hz at wavelengths 750 nm and 820 nm. The fNIRS cap has 16 channels created by eight LED light sources and 8 detectors placed according to the standard 10–20 system, with a source-detector separation distance of 3 cm. Additional short-separation optodes with a distance of 0.8 cm (NIRx Medizintechnik GmbH, Berlin, Germany) to account for superficial blood flow (Yücel et al., 2015) will also be used. We chose to measure cerebral blood flow in the prefrontal cortex region as working memory tasks (such as the n-back) predominately activate this region (Smith and Jonides, 1997; Owen et al., 2005). System calibration will be performed before each assessment using NIRStar 15.2 software and using the predefined montage, and the fNIRS signals will be visually quality checked during data collection for motion artifacts. Cerebral blood flow measures will be taken simultaneously while the participant performs three cognitive tasks of increasing workload (numerical 1-, 2-, and 3-back tests). Our primary outcome variables will be changes in oxy-Hb between the 1-back and 2-back and between the 1-back and 3-back tests (expressed in ΔμM). We will also record deoxygenated (deoxy-Hb) concentrations. Further details about system calibration and data processing are included in Supplementary Material.

#### Cognitive Performance

Cognitive performance in working memory will be assessed using a computerized n-back test (Kirchner, 1958), consisting of the 1-, 2-, and 3-back tests administered simultaneously with fNIRS measures. We will use a computerized, numerical version of the n-back, whereby participants will be required to indicate (*via* a keypress within 2 s from stimulus onset) whether the digit presented on the screen was the same as the digit presented 1 stimulus previously (1-back), 2 stimuli previously (2-back), or 3 stimuli previously (3-back). Each digit will be presented for 1.5 s at an interstimulus interval of 500 ms. The n-back tests were created using E-Prime 2.0 (Psychology Software Tools). To reduce practice effects, participants will perform practice tests before data collection on each experimental day. The outcome variables for cognitive performance will include average reaction time (ms) and accuracy (average number of correct responses) for each test across two blocks of 20 digit sequences (1-back) or four blocks of 20 digit sequences (2- and 3-back).

#### Arterial Stiffness

Arterial stiffness will be measured using SphygmoCor Technology (Butlin and Qasem, 2017). The technology is a non-invasive, reproducible, and accurate method that uses waveform shape parameters from the radial artery to acquire central aortic blood pressure waveform. The aortic pressure waveform will be derived from the radial waveform by a validated transfer function (Pauca et al., 2001). Briefly, after 5 min of supine rest (only at pre-test), high fidelity pressure waveforms will be recorded (three times). The measurements will be ended when all quality parameters (pulse height variation, pulse length variation, diastolic variation, and shape deviation) are fulfilled in the SphygmoCor equipment (SphygmoCor, AtcorMedical, Sydney, Australia; Nelson et al., 2010). The variable for arterial stiffness will be the average of the augmentation index (AIx) across the three measures.

### Other Measures

*Stress* will be measured using salivary cortisol concentrations. Saliva samples (collected immediately on waking on testing days and immediately pre- and post-testing) will be centrifuged and frozen at −80° Celsius and concentrations measured using the ELISA kit Abcam, ab154996. *Mood* will be assessed using the Positive and Negative Affect Scale (PANAS; Crawford and Henry, 2004). *Alertness* will be measured using a simple visual analog scale (VAS; Monk, 1989). *Sleepiness* will be measured using the Karolinska Sleepiness Questionnaire (Putilov and Donskaya, 2013). *Blood glucose* will be collected continuously using an Abbott FreeStyle Libre system (Abbott Scandinavia AB, Abbott Diabetes Care, Solna), which records glucose concentration in the interstitial fluid every minute.

### Sample Size

Several studies (Hyodo et al., 2012; Endo et al., 2013; Byun et al., 2014; Bediz et al., 2016; Carter et al., 2018; Kujach et al., 2018; Ichinose et al., 2020) on the acute effects of light, moderate, and intense physical activity on post-exercise cerebral blood flow have been performed on similar, but not identical, designs and interventions as in our study, but have used comparable populations. Data from these studies were used to recalculate effect sizes using G*Power software (Franz Faul, Universität Kiel, Germany, v 3.1.9.2). Results showed effect sizes between 0.9 and 2.4, rendering a minimum sample size of between 6 and 13 individuals with an *α* = 0.05 and *β* = 0.8 assuming a two-tailed test. We chose to match our sample size to the largest found among the reviewed studies, namely 13 subjects.

### Data Analysis

To answer our primary research question, linear mixed-effects models with subject as the random effect will be used to assess within condition differences in the changes in cerebral blood flow (oxy-Hb) between pre- and post-tests correcting for multiple comparisons from estimation over many channels, using Benjamini–Hochberg. Time and condition interactions will be used to estimate differences between conditions. Baseline arterial stiffness will be assessed as a moderator in the models. Linear mixed models will also be used to assess secondary outcomes, to investigate within-person changes from pre- to post-test, and between conditions. Stata version 15 (StataCorp, College Station, TX, USA) and NIRSToolboxAnalyzer (Santosa et al., 2018) will be used for statistical analyses.

## Discussion

Short, frequent bouts of physical activity may be a promising strategy to increase cerebral blood flow to counteract the negative effects of extended periods of sitting. This crossover randomized trial will determine the effects of frequent, short bouts of physical activity on cerebral blood flow and cognitive performance, as well as psychological factors, and blood glucose levels. This topic is important considering that people are spending increasingly longer periods sedentary each day, particularly in their workplaces (Parry and Straker, 2013), which could potentially lead to long-term unfavorable effects on cognitive function (Voss et al., 2014; Wheeler et al., 2017).

This trial has many strengths. The randomized crossover design reduces contamination and the trial is powered to detect meaningful differences in cerebral blood flow. Importantly, our fNIRS protocol includes short-separation channels to remove superficial blood flow from measures of cerebral blood flow. The use of device-based measures of physical activity, sedentary behavior, and sleep and the use of a VO_2max_ fitness test to personalize the physical activity walking bout are further strengths. The 3-h duration of uninterrupted sitting was chosen as it has high ecological validity; in a cross-sectional study of 334 adults of mean age 42 years, participants rarely sat for longer than 3 h at a time (Bojsen-Moller et al., 2019).

The two exercise paradigms we selected are readily applicable to the workplace setting. If we find immediate effects of physical activity on cerebral blood flow, the next steps would be to develop and test workplace interventions to examine the long-term effects of these paradigms and determine the clinical significance of cerebral blood flow changes on other factors such as productivity, concentration, and cognitive decline.

Finally, this protocol article will contribute to increasing scientific rigor in understanding how immediate, frequent, short activity bouts can affect cerebral blood flow since only one of the previous trials with similar aims has been registered or has published their protocols (Wennberg et al., 2016). Future studies should consider registering and publishing protocols to reduce negative findings not being published and to avoid potential bias of future reviews.

## Author Contributions

ME and ÖE are project leaders. EH, ÖE, OT, MF, and ME were involved in the discussions in designing the protocol and planning the study. EH and CE drafted the manuscript. All authors contributed to reviewing, revising, and approving the final manuscript.

## Conflict of Interest

The authors declare that the research was conducted in the absence of any commercial or financial relationships that could be construed as a potential conflict of interest.
