# Supplementary material for: ABBaH: Activity Breaks for Brain Health. A Protocol for a Randomized Crossover Trial
Source: Front Hum Neurosci. 2020 Jul 14;14:273. doi: 10.3389/fnhum.2020.00273 (PMC7372129; doi:10.3389/fnhum.2020.00273)
Supplement: Supplementary file 1 [file Table_1.docx]

Supplementary Material

1. **Supplementary material – fNIRS calibration and data processing**

FNIRS data will be pre-processed and analysed using the software NIRS ToolboxAnalyzer (Santosa et al., 2018). (<https://github.com/huppertt/nirs-toolbox>). The data is predicted on a block design, which is characterised by alternating periods of activity and rest to facilitate acquisition of the fNIRS signals. Each trial is about 60 seconds in length preceded by a 25 seconds of rest in order to allow the hemodynamic response to go back to baseline and optimise the quality of the response. Raw voltage data will be converted to optical density, then to hemoglobin state concentrations according to the modified Beer-Lambert law. After, the hemodynamic response function will be detected using an autoregressive model based algorithm to solve for the general linear model (GLM), which allows for residuals of the GLM to be at random (Barker et al., 2013). This approach uses pre-whitening filters using autoregressive models and employs iterative reweighted least squares, taking into account serial correlations, motion artefacts, and simultaneous regression of the short-separation channels. Gaussian functions will be used with a set standard deviation and separation of means according to the timeframe of the n-back task for the temporal basis of the function.

**References**

Barker JW, et al. Autoregressive model based algorithm for correcting motion and serially correlated errors in fNIRS. Biomedical optics express. 2013;4:1366-79.

Santosa H, Zhai X, Fishburn F, Huppert T. The NIRS Brain AnalyzIR Toolbox. Algorithms. 2018;11(5):73. PubMed PMID: doi:10.3390/a11050073.

1. **Supplementary material – Fitness testing and ascertainment of speed/inclination of the treadmill for the WALK break condition**

In order to assess *fitness* first a general health screening will be performed, where contraindications for fitness test will be checked, and body mass (in kilograms) and height (in centimetres) will be measured. The participant will be fitted with a heart rate monitor (Polar Oy, Kempere, Finland). Heart rate during the end of the two walking speeds and the end of the maximal treadmill test will be saved.

In a first test, the participant walk at two different speeds and /or inclinations for 4 min each, with one being an easy walk and one a very brisk walk. The heart rate from these two work rates will be used to interpolate or extrapolate the treadmill speed and inclination needed to elicit 75-80% of maximal heart rate. This intensity will later be used as work intensity in the WALK experimental arm.

After the walking test, a 5 min jogging warm up will be performed on the same treadmill, starting at a self-selected speed and inclination. During the warm-up the subject will practice grabbing the treadmill handlebars and exiting the treadmill safely, so that they feel comfortable and acquainted with the stopping technique. At the end of the warm-up, inclination typically will be 1 degree inclination and speed between 8-10 km per hour.

After the warm-up, the participant will be fitted with a face-mask that will be connected to a computerised metabolic system (OxyCon Pro, Erich Jaeger GmbH, Hochenberg, Germany). The system will be calibrated before each measurement. The inspiratory flow meter will be checked with the low and high airflow procedure. The gas analyser calibration will be performed using a gas mixture of 15.0% oxygen and 6.0% carbon dioxide (AirLiquide, Paris, France). During data collection, the breath-by-breath mode will be used.

The maximal treadmill test starts at 1 degree inclination and the same speed as at the end of the warm-up. The maximal test protocols will be individually designed and executed and the speed and/or inclination will be increased every minute until volitional exhaustion. The participants will be given verbal encouragement during the test.

Oxygen consumption (VO_2_) data will be averaged over 10 epochs. Criterion for achieving VO_2_ peak will be a levelling off in oxygen consumption despite an increase in elevation and/or speed. The mean of the three highest 10 s periods will be saved as the peak VO_2_ expressed both as absolute terms (L oxygen consumed per minute, L x min^-1^) and relative terms (mL oxygen consumed per minute and kilograms of body mass, mL x min^-1^ x kg^-1^).
